# Prokaryotic expression of MLAA-34 and generation of a novel human ScFv against MLAA-34 by phage display technology

**DOI:** 10.18632/oncotarget.16590

**Published:** 2017-03-28

**Authors:** Yang Zhang, Pengyu Zhang, Aili He, Yun Yang, Jianli Wang, Hui Zhang, Wanggang Zhang

**Affiliations:** ^1^ Department of Hematology, The Second Affiliated Hospital of Xi'an Jiaotong University, Xi'an, Shaanxi 710004, P.R. China

**Keywords:** MLAA-34, acute monocytic leukemia, phage antibody library, single chain antibody fragment, antibody-based therapy

## Abstract

MLAA-34 is a newly identified monocytic leukemia-associated antigen that is overexpressed in acute monocytic leukemia specifically, thus providing a novel target for the therapy of acute monocytic leukemia. In this study, we first expressed MLAA-34 protein in *Escherichia coli* (*E.coli*) BL21 (DE3) cells and purified it by nickel ion affinity chromatography with high purity (>90%). Then, MLAA-34 was used as antigen for biopanning anti-MLAA-34 single chain antibody fragment (ScFv) from a fully human ScFv library, and a high affinity ScFv named MA1 was selected by phage-ELISA. Finally, after expression of MA1, we found that MA1 can specifically bind with U937 MLAA-34 positive cells, and the binding affinity of MA1 was at the nanomolar level. Furthermore, inhibition of U937 cell proliferation indicated that the novel antibody MA1 has the potential to be used as a therapeutic agent for acute monocytic leukemia.

## INTRODUCTION

Acute myeloid leukemia (AML) is a common malignant disease of the blood and has rapid progression, poor prognosis, and high mortality [1]. Despite intensive induction therapy, where 70-80% of patients with AML achieve complete remission (CR), most patients eventually relapse and die of the disease [2]. Acute monocytic leukemia (AML-M5), as defined by the French-American-British criteria, is a distinct subtype of AML with characteristic clinical features [3]. This subtype is most frequently associated with specific chromosomal anomalies, such as t(8;16)(p11;p13), and translocation involving 11q23, including t(9;11)(p22;q23), t(10;11)(p11;q23), and t(11;19)(q23;p13) [4–6]. Clinically, AML-M5 frequently presents with hyperleukocytosis and extramedullary involvement, including in the liver, spleen, lymph nodes, gingiva, skin, eyes, larynx, lung, bladder, meninges, and central nervous system [7], and more FLT3 aberrations were found in patients with AML-M5 than in other subtypes [8]. Together, hyperleukocytosis, extramedullary involvement, and FLT3 aberrations are associated with an unfavorable outcome in AML-M5 patients.

Leukemia-associated antigens (LAAs) are ideal targets for specific immunotherapies in leukemia patients because they have been shown to induce specific T-cell immune responses [9, 10]. Currently, there are various LAAs under study for AML patients, including WT1, FLT3, BCL-2, and Survivin, as these were the most interesting LAAs in the serologic analysis of a recombinant cDNA expression library (SEREX) with screening using sera from AML patients [11]. Monocytic leukemia-associated antigen (MLAA)-34 is a representative new antigen that we have previously applied the method of SEREX on AML-M5 to identify LAAs through reaction with the sera from AML-M5 patients [12]. In our previous study, we found that MLAA-34 is a novel anti-apoptotic factor, and its anti-apoptotic activity occurs through the β-catenin/TCF 4 pathway [13, 14]. MLAA-34 was shown to localize in the cell membrane and cytoplasm by immunohistochemistry and immunofluorescence [15]. In a clinical study, MLAA-34 was over expressed in AML patients, especially in AML-M5 patients, whereas its extremely low in healthy controls [11]. This expression pattern makes MLAA-34 a potential target for the treatment of AML-M5.

In this study, we expressed and purified MLAA-34 in *Escherichia coli* and screened a novel single chain antibody fragment (ScFv) against MLAA-34 from a human synthetic phage antibody library. The binding affinity and specificity of the specific ScFv named MA1 were evaluated. Our results showed that MA1 has the potential to be used in targeted therapy of AML-M5.

## RESULTS

### Construction of the MLAA-34 recombinant expression plasmid

**Figure 1:**
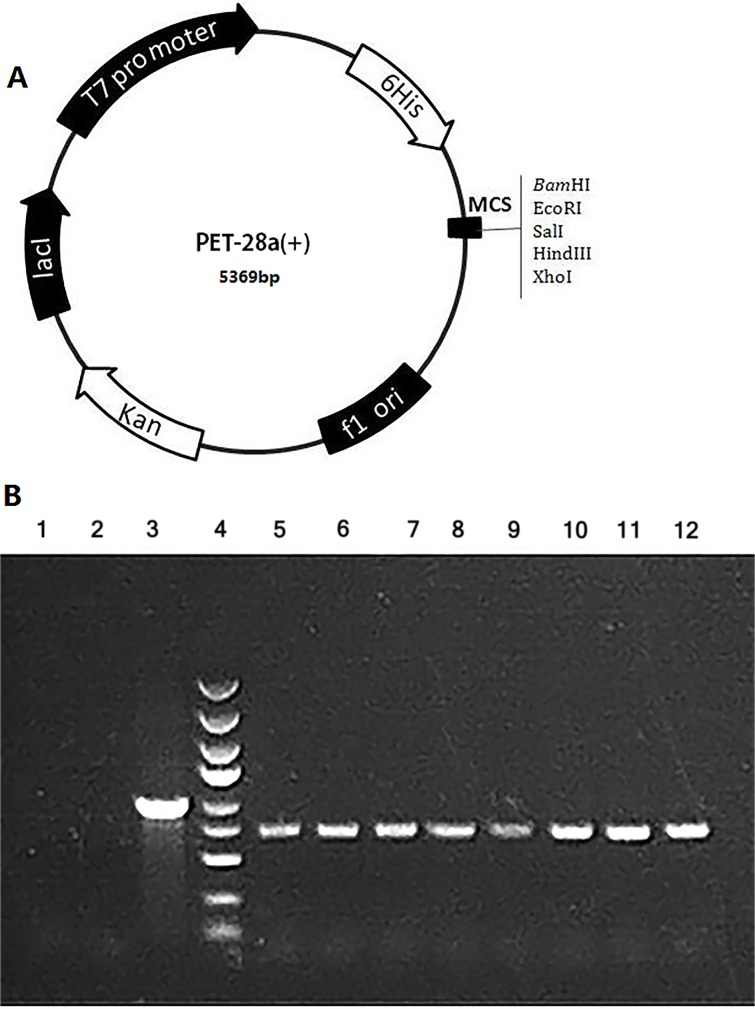
Construction of the MLAA-34 expression vector **(A)** PET 28a Vector information. Component sequence: T7 promoter-6His-MCS Clone sites: EcoRI / SalI. **(B)** The amplified MLAA-34 fragment was cloned into the PET-28a vector, and positive transformation was identified by PCR; the expected product size was 728 bp. Lane 1: negative control (ddH2O); lane 2: negative control (self-connected control group); lane 3: positive control (GAPDH); lane 4: marker 5 kb, 3 kb, 2 kb, 1.5 kb, 1 Kb, 750 bp, 500 bp, 250 bp, 100 bp; lanes 5-12: gene 1-8 transformation.

We constructed a prokaryotic expression vector by cloning the coding sequence of the MLAA-34 gene into the PET-28a(+) vector with EcoRI and SalI (Figure 1A). The positive recombinant clones were identified by PCR (Figure 1B). The insert DNA was confirmed by sequencing.

### Induced expression and purification of MLAA-34 protein

**Figure 2:**
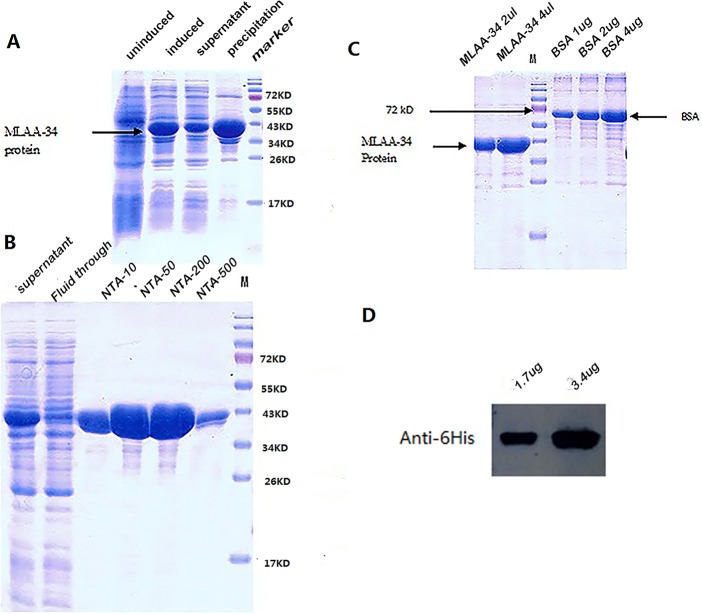
Expression and purification of MLAA-34 in *E.coli* **(A)** SDS-PAGE to show induced and soluble MLAA-34 expression. **(B)** SDS-PAGE to show the purified MLAA-34 protein. *NTA-10/50/200/500 were 10/50/200/500 different concentrations of eluted imidazole. **(C)** SDS-PAGE to show the concentration and purity of the purified protein; the protein concentration was 1.7 mg/ml and the purity was 92%. **(D)** Western blot to identify the purified MLAA-34 protein.

The sonication results showed that MLAA-34 protein was mostly expressed in inclusion bodies, but the soluble portion also produced a significantly induced band near the expected molecular size (Figure 2A). The purification results for MLAA-34 protein indicated that the nickel ion affinity chromatography column was effective, as a high concentration of protein was eluted (Figure 2B). SDS-PAGE was performed using bovine serum albumin (BSA) standards and MLAA-34 protein, and a protein concentration of 1.7 mg/ml and a purity of 92% were obtained for MLAA-34 (Figure 2C). Using the anti-6His antibody, the purified protein was confirmed by western blot (Figure 2D).

### Enrichment, isolation, and identification of high-affinity ScFv

The purified MLAA-34 was used as the antigen, and the ScFv phage clones were enriched by binding to the immobilized antigen, followed by elution and repropagation three times. It was necessary to monitor the titers after each round of biopanning, and the results of the enrichment are shown in Table 1. After three rounds of panning, the titers increased to 10^9^, which indicated an effective enrichment. Among the 188 positive clones, the 50 clones with the highest signals using His-tag as a control antigen were MLAA-34-specific, among which 33 clones were found to be the same as ScFv except spot mutation. The ScFv that had the highest ELISA signal was called MA1, and it was used for further study.

**Table 1:** Biopanning efficacy of phage libraries against MLAA-34

round	Input	Output	Output/Input
1	1×10^12^	3×10^6^	3×10^−6^
2	1×10^12^	2×10^7^	2×10^−5^
3	1×10^12^	3.5×10^9^	3.5×10^−3^

### Expression and purification of MA1

**Figure 3:**
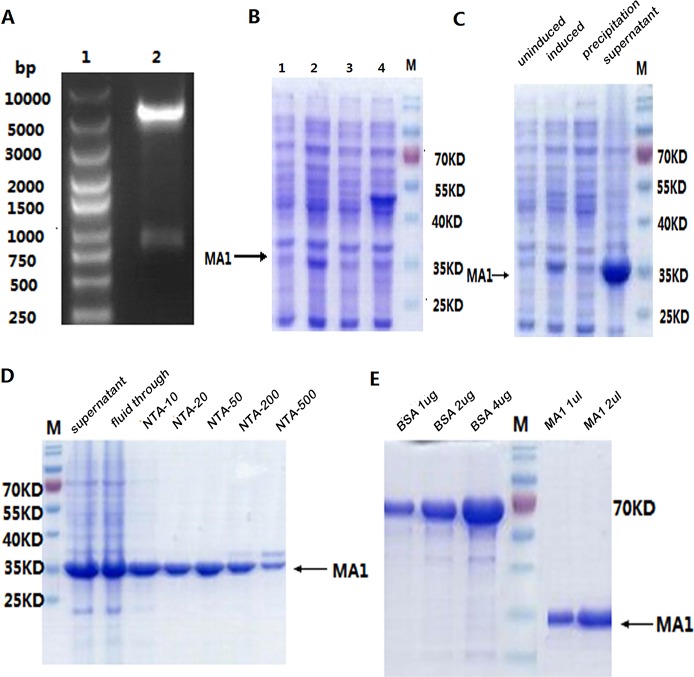
Expression and purification of MA1 in *E.coli* **(A)** Analysis of the plasmid by double digestion. Lane 1: DNA marker; Lane 2: two bands generated by double digestion with BamHI and XhoI. The large band is the vector and the small band is the target DNA MA1 831 bp. **(B)** SDS-PAGE to show MA1 expression condition. Lane 1: total bacterial lysate (not induced); Lane 2: total bacterial lysate (0.5 mM IPTG induced for 4 h, 30°C); Lane 3: total bacterial lysate (0.5 mM IPTG induced for 4 h, 37°C); Lane 4: total bacterial lysate (1 mM IPTG induced for 4 h, 37°C). **(C)** SDS-PAGE to show induced and soluble MA1 expression. **(D)** SDS-PAGE to show purified MA1 protein. *NTA-10/20/50/200/500 were 10/20/50/200/500 different concentrations of the eluted imidazole. **(E)** SDS-PAGE to show the concentration and purity of the purified protein; the protein concentration was 1 mg/ml and the purity was over 95%.

**Figure 4:**
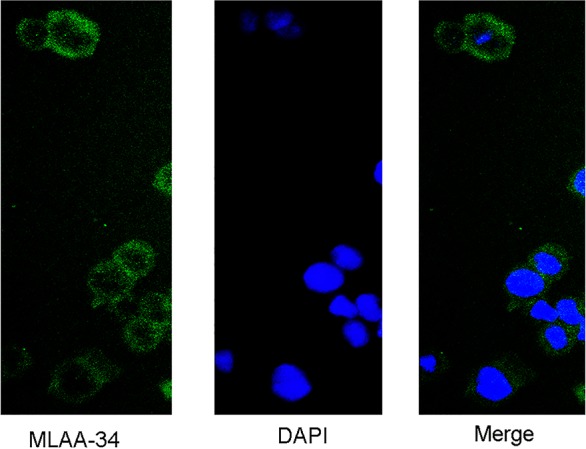
Immunofluorescence staining to show that MA1 can specifically bind to U937 cells in the cytomembrane and cytoplasm (green) The nuclei were stained by DAPI (×40).

We also expressed and purified MA1 in prokaryotic expression system. The recombinant plasmid was digested with BamHI and XhoI (Figure 3A). The insert DNA was confirmed by sequencing. To maximize the amount of soluble protein expression, the conditions we finally choose were IPTG 0.5 mmol/L for 4 h at 30°C. Using 15% SDS-PAGE, we found that the molecular weight of MA1 was consistent with the predicted value 33KD (Figure 3B). The sonication results showed that MA1 protein was mostly expressed in soluble form (Figure 3C). The MA1 was purified with Ni-NTA (Figure 3D). The protein concentration was 1 mg/ml and the purity was over 95% (Figure 3E). Immunofluorescent staining using FITC-anti-6His antibody revealed a fluorescence signal in the cell membrane and cytoplasm of MLAA-34-positive U937 cells (Figure 4).

### MA1 can specifically bind U937 cells

**Figure 5:**
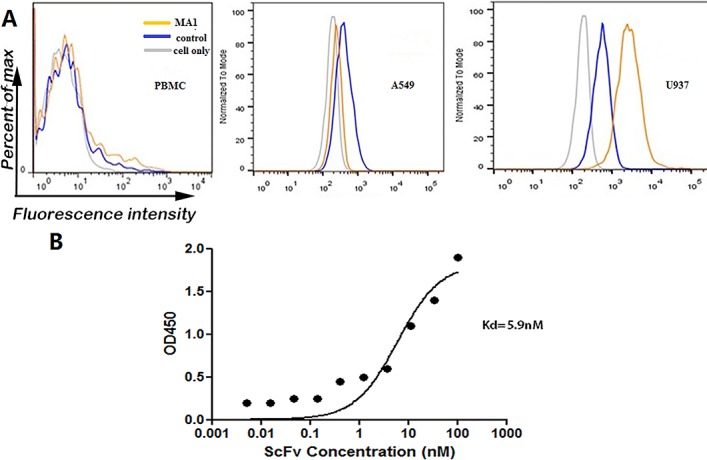
Binding specificity and affinity of MA1 to U937 cells **(A)** Flow cytometric analysis to show binding of MA1 to U937 cells. Anti-TNF-α ScFv was used as negative control. **(B)** ELISA analysis to show the affinity of MA1. The Kd was calculated using nonlinear regression analysis of a one-site binding hyperbola equation in GraphPad Prism 5.0 software.

To evaluate the binding specificity of MA1 with MLAA-34-positive U937 cells, normal human peripheral blood mononuclear cells (PBMCs) and A549 cells were used as the blank control and MLAA-34-negative control. The three types of cells were incubated with MA1 or a negative control anti-TNF-α ScFv, followed by incubation with FITC-conjugated anti-6His-tagged antibody and analysis by FACS. The results showed that MA1 can bind U937 cells, but not A549 cells and normal human PBMCs (Figure 5A). Cellular ELISA was used to evaluate the affinity of MA1, where U937 cells were incubated with MA1. The results showed that MA1 could bind U937 cells at high affinity, with Kd=5.9 nM (Figure 5B).

### Inhibition of MA1 on U937 cells proliferation

**Figure 6:**
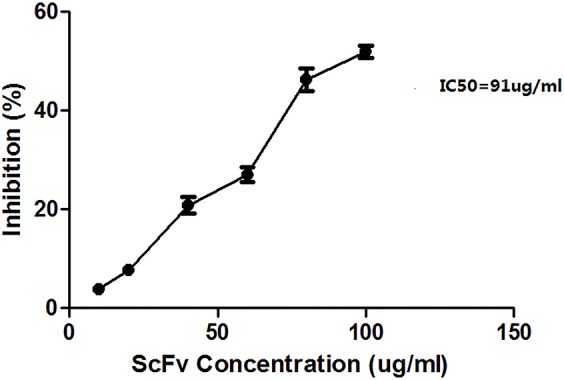
Inhibition of U937 cell proliferation by MA1 The concentrations of MA1 were 10, 20, 40, 60, 80, and 100 μg/ml. Represented data are the mean of triplicate measurements ± SD. The IC50 value was approximately 91 μg/ml.

The inhibition was determined by a CCK-8 experiment, where U937 cells were cultured in different concentrations of MA1. Proliferation was inhibited by MA1, with an IC50 value of approximately 91 μg/ml (Figure 6).

## DISCUSSION

MLAA-34 (GenBank accession no: AY288977.2) is a novel monocytic leukemia-associated antigen that is located on chromosome 13q14.2-14.3. Our previous studies showed that MLAA-34 is an anti-apoptotic factor related closely to carcinogenesis of acute monocytic leukemia [14]. MLAA-34 is localized in the cytoplasm and cell membrane, and it has been identified to be specifically strongly expressed in U937 cells [15]. Thus, it provides an ideal target for AML-M5 therapy.

In this study, we successfully expressed and purified MLAA-34 protein as an immunogen, and it was used to select anti-MLAA-34 antibodies in a phage display antibody library. Antibody-based drug therapy has become one of the most successful and promising strategies for the treatment of cancer. In recent years, many monoclonal antibody drugs have been approved for cancer therapeutic use, like Rituximab and Daratumumab [16, 17]. In current clinical research, more than 500 types of monoclonal antibody drugs are listed, and nearly 50% of them are monoclonal antibody drugs for cancer treatment that act on more than 70 targets [18]. There are also many monoclonal antibodies for AML treatment, and their targets include CD33, CD45, CD66, and leukemic stem cells antigens (CD44, CD123, and CD47) [19–24]. However, the clinical efficacy of these antibodies has not been demonstrated, and most candidate monoclonal antibodies are still under research.

The antibody we prepared was a small molecular antibody. Because MLAA-34 is located in the cytoplasm and cell membrane and intact antibodies could not penetrate the cell membrane. However, small molecular antibodies such as the ScFv antibody have deeper tumor penetration because of their small size [25–27]. ScFv is a fragment of a full antibody in which the variable regions of the heavy chain and light chain are linked to each other by a peptide linker [28]. To obtain ScFv antibodies, we employed phage display antibody library technology, human antibody genes can be fused into phage genes, and the antibodies can be expressed and displayed on the surface of the phages as fusion proteins [27, 29]. The MA1 we obtained was a fully human antibody, murine-origin antibodies are not ideal human therapeutics because of the high probability of developing specific immunity and allergic reactions to the antibodies. Fully human antibodies would be less likely to elicit an immune response and should have a longer half-life *in vivo* [25, 30]. Phage antibody library technique is a new method to obtain fully human antibodies, which are preferred for clinical use because of their negligible immunogenicity [31, 32]. Adalimumab was the first fully human antibody prepared from a phage antibody library, and it was approved by Food and Drug Administration (FDA) for rheumatoid arthritis treatment [33]. The success of adalimumab demonstrated the feasibility of screening human antibodies from a phage antibody library.

In this study, we first expressed MLAA-34 protein in *E. coli* and purified it with Ni-NTA affinity chromatography. This protein was used as an immunogen for screening of its antibody in a phage display antibody library. After biopanning of the ScFv library, we obtained a high-affinity ScFv against MLAA-34, called MA1. ScFvs have a simple structure that allows lower cost when using a prokaryotic expression system [25]. Furthermore, the MA1 binding affinity, specificity, and inhibitory effects of MA1 on proliferation of U937 cells were evaluated. Our results showed that the binding affinity is high, i.e., at the nanomolar level, and MA1 can specifically bind with U937 MLAA-34-positive cells. Furthermore, MA1 can inhibit the proliferation of U937 cells.

In conclusion, we have successfully expressed and purified MLAA-34 protein and isolated a fully human ScFv antibody (MA1) against MLAA-34 from a large human ScFv library. MA1 had good binding affinity and specificity for MLAA-34-positive U937 cells. This work lays a foundation for the development of anti-MLAA-34 antibody drugs. MA1 could be used as a candidate for AML-M5 antibody-based targeted therapy.

## MATERIALS AND METHODS

### Cells and reagents

U937 (human lymphoma monocytic cell line), A549 (human non-small cell lung cancer cell line) and anti-TNF-α ScFv were stored in the hematology laboratory of the 2nd Affiliated Hospital. Healthy human leukocytes were purchased from a blood bank in Xi'an. Cells were maintained in RPMI-1640 medium supplemented with 10% fetal bovine (Gibco) and cultured at 37°C in a 5% CO_2_ humidified incubator. ALPHA™ (Adaptive Library Panning for Human Antibody) is a 8.86*10^10^ human synthetic phage library (EUREKA, China).

### Cloning, expression and purification of MLAA-34

Total mRNA was isolated from U937 cells. The gene of MLAA-34 was amplified with a reverse transcription kit (Boehringer Mannheim, Italy) and cloned into the PMD18-T (TAKARA) vector. The positive clones were confirmed by DNA sequencing, then digested with restriction enzymes EcoRI and SalI (NEB, China), inserted into the PET-28a expression vector (Genechem Company, China), and transformed into a *E. coli* BL21(DE3) (Beyotime Biotechnology, China). The transformed *E. coli* BL21(DE3) was cultured in LB medium containing 50 μg/mL kanamycin and incubated at 37°C and 250 rpm until OD550 of 0.5-1.0. Isopropy-β-D-thiogalactoside (IPTG) was then added to the medium at a final concentration of 1 mM for 6 h. *E. coli* cells were centrifuged at 5000 × g and 4°C for 15 min. Then, lysis Buffer (50mM Tris-HCl, 5mM EDTA, 1%TritonX-100, 1mM PMSF, 0.1mg/ml lysozyme, PH 8.0) was added at 30 mL/g wet weight to re-suspend the *E. coli* cells; cells were disrupted by sonication (200 W power, ultrasonic exposure for 3 sec with breaks of 7 sec, total ultrasonic time of 10 min). The lysate was centrifuged at 5000 × g and 4°C for 30 min. The supernatant and pellet were analyzed using 10% SDS-polyacrylamide gel electrophoresis (PAGE).

The MLAA-34 protein was purified by affinity chromatography using a Ni-NTA column (Merck, Germany) according to the manufacturer's protocol. The purified MLAA-34 protein was separated by SDS-PAGE using bovine serum albumin (BSA) as the standard protein. The brightness of the MLAA-34 bands was compared with the brightness of the band from a known concentration of BSA standard using BandScan software to determine the concentration and purity of the purified protein.

### SDS-PAGE and western blot

Purified MLAA-34 was separated by 10% SDS-PAGE. The gel was stained with Coomassie brilliant blue, and de-stained with destaining buffer (50% (v/v) methanol, 10% (v/v) ethylic acid). For western blot analysis, the protein separated by SDS-PAGE was electroblotted onto polyvinylidene fluoride (PVDF) membrane, then blocked with 5% skim milk in PBST for 2 h at room temperature. The PVDF membrane was incubated with anti-6His antibody primary antibody (1:1000; Abcam, USA) at 4°C overnight. It was then washed and incubated with goat anti-Mouse IgG-HRP (1:2000; Santa Cruz Biotechnology, USA) for 1 h at room temperature. The proteins were visualized using SuperSignal West Pico chemiluminescent substrate (Thermo Scientific, USA).

### Biopanning of the ALPHA phage antibody library

The phage display ScFv library was used for biopanning to isolate anti-MLAA-34 ScFvs using MLAA-34 recombinant protein. All of the manipulations were performed according to Lee [34]. MLAA-34 dissolved in a buffer solution containing 0.1 M NaHCO_3_ was used to coat 96-well plates (Coster, USA) at 25°C for 2 h. The wells were blocked with 5% skim milk at 4°C overnight. Then, the wells were washed two times with PBST (phosphate-buffered saline with 0.05% (v/v) Tween-20). Phages (10^12^ PFU) were added to the wells and incubated for 4 h at 4°C; then, the wells were washed with PBST six times. Then, 0.2 M glycine (pH 2.2) was added to the wells followed by incubation at 25°C for 9 min while rotating on a shaker. Then, the eluate was neutralized with 1 M Tris buffer solution (pH 9.1) in a 2- mL tube and rotated for 10 min. The eluated phages were added to *E. coli* TG1 grown to an OD600 of 0.5. The binding, elution, and infection steps were repeated, and in total, three rounds of biopanning were performed.

### Monoclonal phage-ELISA to identify MLAA-34 specific ScFvs

After three rounds of biopanning, individual phages from the dilution series were tested for antigen binding by monoclonal phage-ELISA. A 96-well plate was coated with MLAA-34 recombinant protein at 4°C overnight, and his-tag was set as negative control. After blocking with 5% (w/v) skim milk at 37°C for 2 h, each well of the plate was washed three times with PBST to remove unbound phages. Then, 100 μL of recombinant phage supernatant was added to each well, followed by incubation at 37°C for 1 h. After washing six times with PBST, the plate was incubated with 1:5000 diluted HRP-conjugated anti-M13 monoclonal antibody in 5% (w/v) skim milk for 1 h at 37°C and detected with TMB substrate. After 15 min, the enzymatic reaction was terminated by adding 50 μL of 1 M H_2_SO_4_, and the absorbance was measured at 450 nm using an ELISA reader. The signal obtained for the negative control should be at least 10- to 20-fold lower than that for the positive control [34]. The positive clones were sent for sequencing to obtain the genes for the ScFvs.

### Expression and purification of ScFv

The ScFv gene which had the highest ELISA signal was cloned into the prokaryotic expression vector PET-28a, and transformed into competent *E. coli* BL21 (DE3) and induced by IPTG, we choose different IPTG concentration and iduced temperature to maximize the amount of soluble protein expression. The ScFv protein was purified by affinity chromatography using a Ni-NTA column (Merck, Germany) according to the manufacturer's protocol.

### Immunofluorescent staining

U937 cells were collected at 1 × 10^6^ /mL, then washed and fixed with 4% paraformaldehyde for 15 min. Cells were incubated with MA1 for 2 h at room temperature and detected by FITC conjugated anti-6His tag antibody (Abcam, USA). After staining with DAPI, the cells were washed, observed, and imaged under fluorescence microscope (Olympus).

### FACS experiment

U937 cells were MLAA-34-positive, A549 cells were MLAA-34-negative, and normal human PBMCs were the balnk control. The cells were washed with PBS and suspended in 5% FBS-PBS (PBS containing 5% fetal bovine serum) at a density of 10^6^ cells/mL. Cells were incubated with MA1 or control ScFv at 4°C for 30 min, followed by incubation with FITC-conjugated anti-6His tag antibody (Abcam, USA) at 4°C for 30 min to 1 h in the dark. The cells were fully dispersed and detected by flow cytometry.

### Cell-based ELISA

To evaluate the binding affinity of MA1 to MLAA-34, MLAA-34 positive U937 cells were grown in 96-well plate overnight and fixed with 4% paraformaldehyde for 10 min at room temperature. Then, they were blocked with 10% goat serum for 30 min at room temperature. MA1 from 100 nM down to 0.005 nM, were added and incubated for 1 h at 37°C. Cells were washed with PBS and incubated with HRP-conjugated mouse anti-6His antibody for 1 h at 37°C and detected with TMB substrate. After 15 min, the enzymatic reaction was terminated by adding 1 M H_2_SO_4_ and the absorbance was measured at 450 nm using an ELISA reader.

### Inhibition of cell proliferation by anti-MLAA-34 ScFv

U937 cells were used to evaluate the inhibition capacity of MA1 with a Cell Counting Kit-8 (Applygen, China). U937 cells (10^5^ per well) in 1640 medium containing 10% FBS were grown in a 96-well plate for 24 h at 37°C in 5% CO_2_, and MA1 was added with different concentrations. Controls included 1640 medium plus U937 cells. Then, WST-8 was added and incubated at 37°C for 1 h, and the absorbance was measured at 450 nm using a microplate reader. The percentage inhibition of cytotoxicity was as follows:

$${\text{Inhibition }(\%)=(\text{OD}}_{\text{control}}-{\text{OD}}_{\text{MA}1})/{\text{OD}}_{\text{control}}\times 100$$
